# Development of mental health first-aid guidelines for suicide risk: a Delphi expert consensus study in Argentina and Chile

**DOI:** 10.1186/s12888-023-05417-0

**Published:** 2023-12-11

**Authors:** Esteban Encina-Zúñiga, Demián Rodante, Martín Agrest, Thamara Tapia-Munoz, Isidora Vidal-Zamora, Sara Ardila-Gómez, Rubén Alvarado, Eduardo A Leiderman, Nicola Reavley

**Affiliations:** 1 Escuela de Salud Pública, Facultad de Medicina, Universidad de Chile, Santiago, Chile; 2 Departamento de Psicología, Facultad de Ciencias Sociales, Universidad de Chile, Santiago, Chile; 3 Facultad de Medicina, Instituto de Farmacología, Universidad de Buenos Aires, Buenos Aires, Argentina; 4 Fundación Foro para la salud mental, Buenos Aires, Argentina; 5 Proyecto Suma, Güemes 4130 (1425), Ciudad Autónoma de Buenos Aires, Argentina; 6 Facultad de Psicología, Universidad de Buenos Aires, Instituto de Investigaciones, Buenos Aires, Argentina; 7 Department of Behavioural Science and Health, University College London, London, UK; 8 Consejo Nacional de Investigaciones Científicas y Técnicas (CONICET), Rosario, Santa Fe, Argentina; 9 Departamento de Salud Pública, Escuela de Medicina, Facultad de Medicina, Universidad de Valparaíso, Valparaíso, Chile; 10 Departamento de Neurociencias, Facultad de Ciencias Sociales, Universidad de Palermo, Buenos Aires, Argentina; 11 Melbourne School of Population and Global Health, Centre for Mental Health, University of Melbourne, Victoria, Australia

**Keywords:** Suicide risk, Mental health first aid (MHFA), Cultural adaptation, Delphi study, Chile, Argentina

## Abstract

**Background:**

Suicide continues to pose a significant global public health challenge and ranks as one of the leading causes of death worldwide. Given the prevalence of suicide risk in the community, there is a significant likelihood of encountering individuals who may be experiencing suicidal thoughts or plans, creating an opening for non-health professionals to offer support. This study aims to culturally adapt the original Australian Mental Health First Aid Guidelines for suicide risk to the Chilean and Argentine context.

**Methods:**

A two-round Delphi expert consensus study was conducted involving two panels, one comprising individuals with personal experience in suicide thoughts/attempts or caregiving for those with such experiences (n = 18), and the other consisting of professionals specialized in suicide assessment and support for individuals at risk (n = 25). They rated a total of 179 items mainly derived from guidelines developed by Australian experts and translated into Spanish (168), and new items included by the research team (11). The panel members were requested to assess each item utilizing a five-point Likert scale. During the second round, items that received moderate approval in the initial round were re-evaluated, and new items suggested by the local experts in the first round were also subjected to evaluation in the next round. Inclusion in the final guidelines required an 80% endorsement as “essential” or “important” from both panels.

**Results:**

Consensus of approval was reached for 189 statements. Among these, 139 statements were derived from the English-language guidelines, while 50 locally generated statements were accepted during the second round. A significant difference from the original guideline was identified concerning the local experts’ reluctance to discuss actions collaboratively with adolescents. Furthermore, the local experts proposed the inclusion of an entirely new section addressing suicide risk in older individuals, particularly focusing on suicide methods and warning signs.

**Conclusions:**

A Delphi expert consensus study was conducted to culturally adapt mental health first aid guidelines for assessing suicide risk in Chile and Argentina. This study involved professionals and individuals with lived experience. While many items were endorsed, some related to inquiring about suicide risk and autonomy, particularly for adolescents, were not. An additional section for older individuals was introduced. Future research should explore the implementation and impact of these adapted guidelines in training courses. This is vital for enhancing mental health support and implementing effective suicide prevention strategies in Chile and Argentina.

**Supplementary Information:**

The online version contains supplementary material available at 10.1186/s12888-023-05417-0.

## Background

Suicide remains a key public health challenge and is a leading cause of mortality worldwide. In 2019, the age-standardized global mortality rate from suicide reached 9.0 deaths per 100,000 population [1]. According to the Global Burden of Disease Study, suicide accounts for 1.34% of total mortality in the general population, and 8% of mortality in individuals aged 10–24, with self-harm ranking as the third leading cause of disability-adjusted life years in this age group [2]. Nevertheless, individuals aged 50–69 (16.2 per 100,000) and 70 years and above (27.4 per 100,000) have higher suicide rates worldwide [1]. Females attempt suicide more, but males have significantly higher suicide death rates [3] In 2019, male suicide mortality was over twofold compared to females (12.6 vs. 5.4 deaths per 100,000 population) [1]. Reasons include lethal methods, lower help-seeking [4] and multiple risk factors like alcohol misuse [5].

Low- and middle-income countries (LMICs) accounted for the majority of suicide deaths (77%) in 2019, but high-income countries (HICs) have the highest age-standardized suicide rate at 10.9 (ASR) per 100,000 [1]. Between 2000 and 2019, while several regions experienced a decline in suicide mortality rates, the Americas observed a significant increase [6]. Despite this, in Chile, the ASR per 100,000 population decreased from 10.5 in 2000 to 8.0 in 2019, with the greatest variation observed in men (from 19.0 in 2000 to 13.4 in 2019). According to the latest Chilean National Health Survey (2016-17), in the previous 12 months, 2.2% of individuals reported suicidal ideation (2.8% females, 1.7% males); 1.5% made a suicide plan (0.4% males, 2.5% females); and 0.7% attempted suicide (0.2% males, 1.3% females) [7]. The analysis by age group shows that individuals over 70 years old exhibit the highest suicide rates, with 24.7, 29.2, and 39.5 per 100,000 inhabitants for the age ranges 70 to 74, 75 to 79, and 80 and above, respectively [8].

Overall, Argentina also showed reduced mortality from suicide, from 9.2 in 2000 to 8.1 in 2019 [1]. Between 1990 and 2019 Argentina registered 85,189 deaths by suicide, with a significant difference between males and females (3.8 deaths by suicide in males compared to females). The analysis by age group shows that in 2018, individuals over 75 years old exhibits highest suicide rates than the average rate, with 11,1 per 100,000 inhabitants for the 75–79 years old group and 11,7 per 100,0000 inhabitants for the 80 years old and above group [9]. Over a 30-year span, mortality rates in Argentina showed three distinct periods: (a) descending rates between 1990 and 1998; (b) ascending rates between 1998 and 2003, being this period marked by the social, political and economic crisis of the time; (c) descending rates from 2003 to 2019 [10]. Despite expectations of rising mortality rates by suicide due to the COVID-19 pandemic, an initial study in the Province of Buenos Aires (Argentina) showed a 23.1% lower number of deaths by suicide for 2020 than expected based on 2015–2019 provincial statistics [11].

Risk factors for suicide behavior include individual psychological traits [12, 13], harmful substance use [14, 15], gambling [16], access to lethal means [17], and physical pain [18]. A history of repetitive self-harm may also increase the risk [19]. Substance misuse, history of self-harm, or suicidal planning can increase the risk of progressing from suicidal ideation to suicide attempt [20]. At a psychosocial level, risk factors for suicide include marital status [14, 21], economic resources [22], low educational attainment [23], alcohol abuse in the household [24], early childhood adversity [25–27], and bereavement [28]. At a community-based level, domestic or neighborhood violence [29, 30], cultural and gender aspects [31, 32], economic crises [33] and stigma have also been studied as factors that increase the risk of suicide [34, 35]. These issues, and the findings that many people who complete suicide have no history of mental illness, have led to growing emphasis on a public health approach to suicide. This recognises that many intrapersonal, interpersonal, community, occupational, environmental, and societal factors contribute to suicide risk [36].

### Variability across different settings and cultures

Given that most of the research has been conducted in HICs, it is crucial to be cautious when extrapolating findings to LMICs populations. Indeed, despite the fact that the majority of suicide deaths occur in LMICs, less than 15% of research is conducted in these regions [37], which could lead to the use of insufficient or inappropriate knowledge [38]. For example, while evidence from HICs emphasizes the importance of treating psychiatric disorders for the prevention of suicide, evidence from LMICs indicates a lower prevalence of psychiatric disorders among individuals who self-harm or die by suicide [39, 40], with some studies suggest that direct cash transfers would be more closely linked with to reducing suicide deaths [41]. Such evidence indicates that, in these contexts, strategies aimed at reducing social and economic inequalities may be more effective in lessening suicide than those focused on treating psychiatric disorders at the population level.

Another example of contextual and cultural differences has been observed in suicide bereavement. Research in HICs has shown that individuals who have experienced a suicide loss may receive a lower degree of support than those bereaved by other causes of death [42]. Given the family structure in LMICs, it is not unreasonable to expect the impact of suicide bereavement may extend to a larger number of people compared to HICs [43]. Nonetheless, the decreased level of support mentioned above may not necessarily be applicable to LMICs, as community-based responses in those contexts could be more resilient compared to HICs. This type of community support could potentially contribute to the lower occurrence of recurrent self-harm episodes (and subsequent suicide mortality) observed in certain LMICs [44], and making it particularly relevant for mental health literacy and first aid strategies, in which are the focus of the current study.

The concept of *familism*, encompassing Latin-American notions of familial obligations, support from family, and family as a reference point, has been suggested to influence suicidal behavior [45]. Conflicts between the values of familism and adolescence can result in reduced mutual support, externalizing behaviors in male adolescents, internalizing behaviors in adolescent females, and ultimately an increased risk of suicide attempts [44]. Gender roles and behaviors associated with *Machismo* in Latin America can also contribute to substantial gender disparities in rates of suicidal behavior among young individuals [46]. Religion, on the other hand, plays a significant role in shaping the perception of suicide within Latin American and Caribbean (LAC) cultures. Catholicism, for instance, often considers suicide as a sinful act deserving punishment and reinforces patriarchal gender roles [47, 48]. Conversely, certain Pagan traditions, like the Mayan belief in Ixtab, a deity guiding suicidal individuals to paradise, may view suicide more positively [49].

In ethnic, sexual and gender minority populations, studies show that cultural factors, encompassing aspects such as discrimination, acculturative stress, family conflict, social discord, and cultural norms, demonstrate a comparable predictive value to conventional factors such as hopelessness, depression, and reasons for living, which are commonly incorporated in conventional risk assessment protocols [50]. Taken into consideration as a whole, these studies expand and strengthen research by demonstrating that cultural factors have a significant impact on the prediction of suicidal behaviors [51, 52], demonstrate the complexity involved in suicidal behavior and reveal its contextual predictive value [53].

### Mental health services for suicide risk in Chile and Argentina

Chile has included suicide prevention strategies in its policies since the 1990s [54–57], but it was not until 2013 that the *Plan Nacional de Prevención del Suicidio* was formulated [58], integrating different health actions with intersectoral efforts. The plan was piloted first in three regions and consisted of six components, including (a) case study system installation, (b) intersectoral regional suicide prevention plan implementation, (c) professional health workers’ competency strengthening, (d) preventive programs in educational establishments, (e) crisis support system development, (f) and technical support for appropriate media coverage and reinforcement of the role of media in suicide prevention. In 2020, the program was evaluated by government agencies [59] and expanded to the rest of the country but shifting the strategy towards capacity-building for suicide prevention among healthcare personnel, educators, and the general community. In 2022, Chile launched a dedicated suicide prevention hotline that operates 24/7.

In Argentina, a national law for suicide prevention was enacted in 2015 (regulated in 2021 and adhered to by all 24 jurisdictions) and aims to foster biopsychosocial care, advance scientific and epidemiological research, provide professional training for identifying and assisting individuals at risk of suicide, and offer support to families affected by suicide [60]. According to the national law 27,130, one of its aims is the promotion of supporting networks within the civil society for the purposes of prevention, detection of people at risk, treatment, and training.

Overall, suicide prevention policies in Latin America share the common element of increasing awareness through public education, improving societal attitudes and beliefs, and eliminating stigma towards individuals with mental disorders or suicidal behavior [61].

### Community-based support strategies and mental health first aid

In recognition of the need for a public health approach to suicide, it increasingly recognised that effective suicide prevention requires action within and outside of the healthcare sector, as represented by a Balanced Care Model [62] that combines services which gradually shift from hospital-based care to specialized outpatient community services, primary health care, intersectoral actions, informal community care and self-management of mental health [63]. Studies have shown that community involvement improves health outcomes [64] and has the potential to reduce the mental health treatment gap by improving the health workforce (e.g. community health workers), customizing health programs to address specific local needs and resources (e.g. through health service user councils) and by increasing mental health literacy, reducing stigma, improving help-seeking behavior and promoting self-managed care of mental health [65, 66].

To cover the base of the pyramid of balanced services, the role of the community is irreplaceable, requiring participation both in the management of services and in the actions of supports groups and self-care [67, 68]. In this context, the training of community mental health workers (also known as task-sharing) is a strategy with increasing evidence of its effectiveness in promoting and providing mental health care [69, 70]. These approaches have been incorporated into the recommendation of both the latest World Mental Health Report 2022 [71] and the Mental Health Action Plan 2010–2030 [72], published by the World Health Organization.

Chile has had various experiences in community capacity building throughout its history. The Intracommunity Psychiatry Program in the 1970s aimed to train community members together with other education and health professionals to generate a stepped system of task delegation [73]. Similar ideas have garnered renewed interest in recent years [74], converging with international strategies such as the Mental Health Global Action Programme (mhGAP) [75] for training non-specialist health personnel, which has recently expanded its scope to include lay community members [76].

In Argentina, the CAS (Centro de Asistencia al Suicida [Suicide Assistance Center]), a non-government organization located in Buenos Aires, was created in 1967 and has, since then, offered a telephone helpline and other resources for the person at suicide risk, friends, and family, and more recently displayed on their webpage https://www.asistenciaalsuicida.org.ar. Fortunately, in recent years the National Ministry of Health of Argentina and other provincial ministries, in the context of the Federal Strategy for a Comprehensive Approach to Mental Health, have set up other free 24-hour lines for accompaniment, support and guidance on mental health [77].

Globally, Mental Health First Aid (MHFA) training is an approach aimed at improving the general population’s mental health literacy and providing early intervention within a community setting, where access to health services might be limited [78]. Given the prevalence of suicide risk in the community, there is a significant likelihood of encountering individuals exhibiting symptoms, creating an opening for non-health professionals to offer support. Considering this, Kitchener and Jorm [79, 80] created the MHFA training programs to teach individuals how to effectively support someone with a developing mental health problem or who is in a mental health crisis. The training includes an action plan comprising the following steps: (1) Risk assessment; (2) Non-judgmental listening; (3) Reassurance and provision of information; (4) Encouragement to seek appropriate professional assistance; and (5) Encouragement of self-help and other support methods. The aid is extended until the crisis is resolved, professional assistance becomes accessible, or the situation is resolved. The dissemination of MHFA has been extensive, reaching more than 25 countries, but most of them are HICs. A systematic review and meta-analysis of randomized controlled trials of MHFA training conducted in 2018 revealed significant reductions in stigma, enhancement in mental health literacy, and helping behavior that persisted for up to six months post-training [65].

Drawing on previous studies of cultural adaptation of mental health first aid guidelines for suicide risk for China [81], Sri Lanka [82] and Brazil [83], a consortium of Chilean, Argentinian and Australian global mental health researchers and clinicians used the Delphi expert consensus methodology to culturally adapt guidelines to train lay members of the community and non-specialized health care providers interested in providing mental health first aid to someone at risk of suicide in Chile and Argentina.

## Methods

This study is similar to other Delphi studies conducted by our team to culturally adapt the MHFA guidelines to Chile and Argentina [84, 85] and by those of the broader research group to adapt guidelines in several other countries [81–83, 86, 87]. It comprised four stages: [1] The development of the first-round survey; [2] Expert panel member recruitment; [3] Data collection and analyses for the round 1 and 2 surveys; and [4] Guidelines development.

### Round 1 survey development

The initial round of the questionnaire was crafted through the translation of statements derived from mental health first aid guidelines utilized in English-speaking countries to support a person at risk of suicide [88, 89]. The 168 items from the English guidelines were translated into Spanish and reviewed by bilingual mental health professionals from Australia, Argentina, and Chile to ensure a culturally appropriate and accurate translation. Three of these items were reformulated based on the assumption of the research team that they required tailoring to the local context. An additional 11 items were incorporated on the recommendations of the research team.

The original survey was composed of eight sections: [1] Identification of suicide risk (29 items), which included items related to approaching someone at suicide risk, asking about and reacting to suicidal thoughts, special cases (e.g., the person with psychotic symptoms or under the influence of drugs or alcohol); [2] Assessing the seriousness of the suicide risk (18 items), which included assessing urgency, finding out about a plan and other factors contributing to suicide risk; [3] Initial assistance (35 items), which included items about practical situations (e.g., when the person has a plan and the means to carry out their suicide plan, the person is reluctant to give the first aider the things they intend using to kill themselves, or has attempted suicide in the past; [4] Talking with a suicidal person (40 items), which included items about letting the person know the first aider cares about them, active listening, highlighting positive factors, and what to avoid when talking with a suicidal person; [5] Safety planning (8 items), which included items about the components and characteristics of a safety plan; [6] What the first aider should know (15 items), which included items about the suicide risk, the suicidal person and local mental health resources; [7] Confidentiality (7 items), which included items about not accepting secrecy for a suicide plan and what to do when the suicidal person refuses to give permission to disclose information about their suicidal thoughts; and [8] Adolescent specific items (27 items), which included items about involving family or others, helping the adolescent to look for help, and involving the adolescent in decisions about their safety.

### Expert panel member recruitment

Members of the expert panels were recruited by six members of the research team (MA, EL and SAG, Argentina; EE, IZ and TT, Chile). One panel comprised experts with lived experience, either their own or as an informal caregiver (e.g., family or friends of a person who experienced a suicidal crisis). Panel two comprised health professional experts. Lived experience experts were only recruited in Chile and included participants who received treatment in health services for their suicide risk or for a suicide attempt in the past, and/or their caregivers, although they were not recruited through the mental healthcare services. Health professionals included members of different health disciplines who worked as health providers (e.g., clinical psychologists, psychiatrists, and primary care workers) as well as researchers and decision-makers.

The invitation to recruit participants was a very close translation from the original Australian version. Participants were asked for their views on actions related to how to help someone who is experiencing a mental health problem or is in a mental health crisis (“*brindar sus opiniones sobre las acciones relacionadas con la forma de ayudar a alguien que está desarrollando un problema de salud mental o que se encuentra en una crisis de salud mental* “). A broad definition of “person who may be experiencing suicide risk” (“*persona que puede estar experimentando riesgo de suicidio*”) was adopted, without further specification. Recruitment was done by snowballing and by digital posters on the participating universities’ social networks. As with our previous studies, the following criteria had to be met for a person to be eligible for the study:

- For the professional expert panel: more than four years of experience working as a health care professional with expertise in suicide assessment and helping people with suicide risk. Eligible types of professions included: general practitioners, nurses, occupational therapists, psychiatrists, psychologists and social psychologists.
- For the lived experience expert panel: self-identified as having experience with suicide thoughts and/or suicide attempts or caring for a person with past experiences of suicide risk.
- For both panels, aged 18 years old and above.

This study was developed during the Covid-19 pandemic, so participants provided informed consent by email or by WhatsApp (a free US platform widely used in Latin America for instant messaging between cell phones). They signed the informed consent form along with a signature of a witness and sent a photo of the signed document.

### Data collection and analysis

Data for the first round was produced between March 21, 2020, and January 18, 2022. Data for the second round was collected between September 2, 2022, and November 23, 2022. Surveys were conducted online through *Qualtrics* software for both rounds.

The participants rated a set of statements on a 5-point Likert scale (1 = essential, 2 = important, 3 = unsure, 4 = not important, 5 = should not be included), selecting how important they considered the inclusion of each statement in the final mental health first aid guidelines for suicide risk in Argentina and Chile.

Items were immediately accepted for inclusion in the final guideline if at least 80% of the participants in both panels rated it as “essential” or “important” in the first round. Meanwhile, statements rated as “essential” or “important” by at least 70.0–79.9% of both panels in the Round 1 survey were re-rated in Round 2. Statements rated as “essential” or “important” by less than 70% of participants from at least one panel were immediately excluded from the final guidelines. In Round 2, recommendations with an acceptance rate of at least 80% by one panel and at least 75% by the other panel were selected for the final guidelines.

At the conclusion of each subsection or following every set of 10 items (whichever occurred first), participants were provided with open-text response boxes. This allowed them to share their comments or propose fresh recommendations they believed should be integrated into the final guidelines. MA and EE formulated novel items based on these suggestions garnered from the initial round. Prior to their inclusion in the second round, these newly created items underwent a discussion with NR. This discussion ensured their feasibility, applicability, and distinctiveness from the items already presented. Furthermore, items that did not attain a minimum of 80% support but were subject to suggestions for improved language or the necessity for clarification were revised and subsequently included in the second round for re-evaluation Some items (n = 4) were presented in the second round with two different formulations (the originally evaluated in the first round and a rephrasing suggested by one or more experts).

Spearman’s correlation coefficient was estimated for the association analysis between the approval ratings of the professional and lived experience panels. SPSS version 25 software was used.

### Guidelines development for Chile and Argentina

EE, DR, and MA consolidated the recommendations from the two rounds of surveys into a preliminary guideline document. The entire team reviewed the draft and provided input for a new version. The document was also sent to a small number of participants who expressed special interest in reviewing the draft guidelines. As a result of their feedback, some minor changes were made.

### Ethical approval

The study received ethical approval from the University of Melbourne (in Australia), the University of Palermo (Argentina) and the University of Chile (Chile).

## Results

**Fig. 1:**
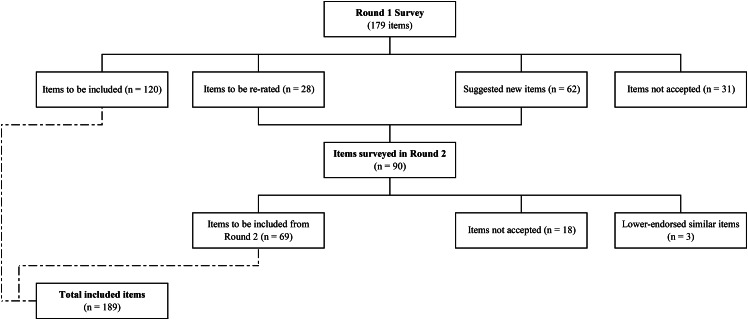
Statements included, re-rated, and excluded in the two survey rounds

Figure 1 shows the overall process of including the statements through the two rounds.

### Round 1

We received a total of 49 answers for the Round 1 questionnaire. The professional panel (n = 27) included more answers from Argentina (n = 16) than Chile (n = 11) and included 13 psychologists, 12 psychiatrists, one general practitioner, and one nurse. The average years of experience as a health professional was 18.8 years, with 51.9% females (n = 14) and 48.1% males (n = 13).

The lived experience panel (n = 22) exclusively included participants from Chile. This was due to logistical considerations associated with a broader Mental Health First Aid study conducted in these countries. Among the participants, twenty were consumers, while two were informal caregivers and/or relatives. Among those who identified their main role as consumers, two were also healthcare workers. Similarly, among those who primarily identified as caregivers, one individual was also a healthcare worker. The panel comprised 72.7% females (n = 16) and 27.3% males (n = 6). See Table 1 for a summary of the sociodemographic characteristics of participants.

Out of the 179 items that were part of the Round 1 survey, a total of 120 items (67.0%) garnered endorsement as being essential or significant by 80% or more of the experts in both panels. An additional 28 items (15.6%) required re-evaluation in Round 2, while 31 items (17.3%) were rejected (Fig. 1). The overall endorsement rates were 73.2% for the lived experience panel and 74.9% for the health professional panel, indicating a preliminary level of consistency. Only five items (2.8%) were endorsed by one panel and rejected by the other, signifying a high level of agreement between the two panels.

**Table 1:** Sociodemographic characteristics of participants

Lived Experience	Round 1				Round 2			
	Argentina		Chile		Argentina		Chile	
	n	%	n	%	n	%	n	%
**Gender**								
Female	-	-	16	72,7	-	-	13	72,2
Male	-	-	6	27,3	-	-	5	27,8
Other	-	-	0	0	-	-	0	0
Total			22	100			18	100
**Type of LE**								0
Consumers	-	-	20	90,9	-	-	17	94,4
Caregivers	-	-	2	9,1	-	-	1	5,6
Total			22	100			18	100
**Age**								
18–24	-	-	8	36,4	-	-	2	11,1
25–34	-	-	11	50	-	-	12	66,7
35–44	-	-	2	9,1	-	-	3	16,7
45–54	-	-	0	0	-	-	0	0
55–64	-	-	1	4,5	-	-	1	5,6
65+	-	-	0	0	-	-	0	0
Total			22	100			18	100
**Total**	**22**				**18**			
**Professionals**	**Round 1**				**Round 2**			
	**Argentina**		**Chile**		**Argentina**		**Chile**	
	**n**	**%**	**n**	**%**	**n**	**%**	**n**	**%**
**Gender**								
Female	5	31,3	9	81,8	5	33,3	8	80
Male	11	68,8	2	18,2	10	66,7	1	10
Other	0	0			0	0	1	10
Total	16	100	11		15	100	10	100
**Age**								
25–34	1	6,3	5	45,5	0	0	6	60
35–44	4	25	4	36,4	5	33,3	3	30
45–54	7	43,8	2	18,2	2	13,3	1	10
55–64	3	18,8	0	0	7	46,7	0	0
65+	1	6,3	0	0	1	6,7	0	0
Total	16	100	11	100	15	100	10	100
**Profession**								
Psychologist	4	25	8	72,7	3	20	7	70
Psychiatrist	12	75	0	0	12	80	0	0
Nurse	0	0	1	9,1	0	0	0	0
General Practitioner	0	0	1	9,1	0	0	1	10
Other	0	0	1	9,1	0	0	1	10
Total	16	100	11	100	15	100	10	100
**Total**	**27**				**25**			

### Round 2

The Round 2 questionnaire included 28 items to be re-rated and 62 new items that were suggested in Round 1 (Fig. 1). A total of 43 answers were received in Round 2, with 25 from the health professional panel (response rate of 92.6%) and 18 from the lived experience panel (response rate of 81.8%). Out of the 90 items rated in Round 2, 76.7% (n = 69) were endorsed by both panels and 20.0% (n = 18) were rejected. An additional 3.3% (n = 3) were items that were endorsed but a different formulation of the same items had greater acceptance and were thus not included in the guidelines.

### Differences between the spanish-language guidelines for Chile and Argentina and the english-language guidelines

In total, considering both rounds, a total of 189 items received endorsement, while 52 items were rejected. It was observed that 37 statements (20.7%) present in the English guidelines did not find acceptance among the Argentinian and Chilean experts. All 11 items incorporated in Round 1 by the research team (plus the three reformulated items) were endorsed by both panels. Among the 62 new items suggested by the local experts for Round 2, the proportion of rejected items (19.4%) was comparable to the percentage of original items rejected. Overall, 50 new items suggested by the local experts were added.

Rejected statements covered:

- How to address and communicate with the person at suicide risk (e.g., The first aider should know that it is more important to ask about suicidal thoughts than to be concerned about the exact wording; The first-aider should avoid using terms to describe suicide that promote stigmatizing attitudes, e.g. ‘commit suicide’ or refer to a suicide attempt as having ‘failed’ or been ‘unsuccessful’; The first aider should only ask about issues that affect the immediate safety of the person who is suicidal);
- Offering reassurance, normalizing suicide thoughts and highlighting positive factors (e.g., The first aider should reassure the suicidal person that thoughts of suicide are common, and that many people have them at some stage in their lives; The first aider should remind the suicidal person that suicidal thoughts need not be acted on; The fact that the suicidal person is still alive, and talking to the first aider about their feelings, means that they are not quite sure about suicide. The first aider should point this out as a positive thing);
- When to involve the police in a suicidal crisis (e.g., The first aider should contact the police if the suicidal person has a weapon);
- When communicating with an adolescent, the first aider should refrain from providing advice and, in certain circumstances, should avoid entering into a dialogue concerning the specific actions the adolescent should undertake to seek assistance.

The items that received the highest average rejection from both panels were: (a) The first aider should solely ask about matters that directly impact the immediate safety of the individual (with 37.1% approval); (b) When engaging in dialogue with an adolescent, the first aider should refrain from providing advice (48.7%); and (c) The first aider should inquire whether the individual has ever known someone who died by suicide (55.1%).

On the other hand, during round 1, the local experts suggested the inclusion of a completely new section on suicide risk in older people. All ten items proposed for the section were endorsed in round 2 by both panels including: (a) What the first aider should know about suicide methods in this population (e.g., greater lethality of suicide attempts, passive methods that can cause their death); (b) Warning signs indicating a greater risk of suicide attempt (e.g., increasing isolation during the last six months, increasing helplessness, loss of autonomy, difficulties handling physical pain; receiving a disturbing medical diagnosis).

Other items suggested by Chilean and Argentinian experts that were accepted in the second round included aspects related to the identification of suicide risk (e.g., The first aider should be aware that if a person has suicidal tendencies, asking them about suicidal ideas can reduce the risk and be an opportunity to find a better and more effective solution to the problem that causes their suffering; The first aider must be aware that a person who is seriously thinking about suicide will not always say so, and could even appear calm).

The importance of reaching out to the personal network of someone at suicide risk and contacting healthcare services that could offer support were highlighted by both panels. With regards to the initial assistance, local experts suggested and endorsed for incorporation in the guidelines the following items: Although it is important to show calm, the first aider must be aware that in the face of a high risk of suicide they must act quickly to activate the support network and/or the appropriate specialized services; The first aider must consider that the person may encounter obstacles in accessing health services in relation to the risk of suicide and accompany the person to overcome them; If the person at risk of suicide is in a confrontational situation with the first aider’s suggestions, the first aider should give priority to empathy and support over persuasion.

See supplementary file 1 for a full list of the statements excluded from the original guidelines and new items added in the final guidelines.

### Differences between the lived experience and health professional panels

Over both rounds, the level of agreement between panels was high (with Spearman r = 0.72 in Round 1 and r = 0.62 in Round 2). During the first round, for 75.0% of the statements (n = 135) there was less than a 10% difference in the percentage of panel members endorsing those items, including 5.0% (n = 9) of the items with complete agreement in both panels (i.e., 100% of the members of both panels endorsing the item). On the other hand, there were 6.1% of items (n = 11) where disagreement between panels was 20% or greater and only 0.56% of items with disagreement greater than 30% (n = 1). Items endorsed by one panel (80% agreement or greater) and rejected by the other panel (less than 70% agreement) totaled 2.6% of total items in Round 1 (n = 5).

In Round 2, 63.3% of items had less than 10% difference in the percentage of members of panels endorsing those items, including 10.0% of items (n = 9) with absolute agreement. Disagreements of 20% or more were found in 7.8% of items, (n = 7), including 2.2% of items with a 30% difference or greater (n = 2).

The greatest differences in Round 1 included items related to directly asking the suicidal person if they have a suicide plan, and how, when and where they are planning on doing it. Experts from the professional panel mostly endorsed this way of asking (85%), while half the experts from the lived experience panel did not endorse using direct questions about suicide plans. Interestingly, both panels agreed that asking someone about their suicidal thoughts or plans would not increase the probability of the suicidal person acting upon them (and would in fact allow other solutions to their suffering).

There were also notable differences with regards to asking about specific situations that may be causing suicidal thoughts. While lived experience experts endorsed asking about situations of discrimination or social or work/educational abuse that could be related to suicidal thoughts, only 60% of the health professional panel endorsed this statement. The lived experience panel unanimously endorsed that “The first aider should not mention the instances of help that are more difficult to accept from the outset (for example, hospitalizations, emergency interventions), but should take them into account to mention them at the right time and with words that do not generate rejection by the suicidal person.” However, barely 65% of health professionals endorsed this statement and it was rejected.

See supplementary file 1 for details of the ratings of statements by round and panel, and for the final guidelines text in Spanish.

## Discussion

The current study utilized the Delphi expert consensus approach to culturally tailor guidelines for community members who wish to offer mental health first aid to individuals at risk of suicide in Chile and Argentina. This was achieved through a two-round Delphi survey that engaged mental health professionals, individuals with personal lived experiences, and informal caregivers. The final guidelines comprised 189 statements endorsed by both panels.

### Contrasting perspectives of health professionals and lived experience experts on first aid for suicide risk

The responses of health professionals and experts with lived experience were highly correlated with regards to their evaluation of most items, particularly in the first round. However, while professionals agreed that the first aider should be aware that directly asking about suicide does not increase the risk (and can provide an opportunity for exploring other solutions), and that they should inquire about such thoughts and plans, lived experience experts only endorsed the awareness aspect but did not fully support the act of asking. This apparent inconsistency could be interpreted as a manifestation of concern or insecurity on the part of the lived experience experts, perceiving that they might ask inappropriate questions and generate undesirable effects, such as a sense of mistrust from the person, which could create barriers to disclosing suicidal thoughts [90, 91].

In contrast, the item relating to asking the person about other things in their personal life (e.g., situations of discrimination or social or work/educational abuse) that might be affecting them was endorsed by the lived experience experts but not the professional panel. The professional panel rejected going beyond the suicidal situation and recommended that the first aider stick to the crisis resolution and refer to additional sources of help. This difference may point to people with lived experience focusing more on the factors leading to a person’s suicidality, while health professionals (particularly those trained in psychology) may have focused more on the psychological processes of a person considering suicide. Health professionals may also have considered psychosocial factors contributed to risk were less relevant to their decisions on how to help the person at immediate risk, or that such considerations may open the door to a conversation that might be beyond the first aider’s capacity to manage. Nevertheless, as pointed out by Chu et al. there is evidence that these psychosocial factors are significant predictors of suicidal behavior [50]. Hence, the limited emphasis given by the professional panel to these factors may necessitate a more critical examination of disciplinary practices.

Interestingly, this is partially consistent with findings from other recently adapted MHFA guidelines in Chile and Argentina, where attention to social determinants—in terms of risk exposure or addressing vulnerable groups—seems to be more relevant to the lived experience experts [85]. This wider view of suicide risk is more in line with a public health approach [36].

### Comparison with the guidelines for english-speaking countries

A significant difference to the original guidelines was seen in items about directly discussing or inquiring about suicide. Rejected items included those relating to asking about suicidal thoughts or plans, while newly added items highlighted positive effects of asking about suicide such as providing an opportunity to explore more effective and comprehensive solutions to the problem causing distress. The disapproval of inquiring about suicide or its plans may have been more likely to be endorsed in circumstances where the person helping had greater expertise, a scenario that is more likely after training [92]. Similarly, the expression of feelings by the first aider was not supported by the health professional panel. By rejecting statements indicating that the helper should express their own concerns or gratitude for the person sharing their feelings, it is possible that the experts were concerned about the first aider making overly personal comments that may trivialize the individual’s problem or bias the assistance by including the helper’s own experiences.

Another area of significant differences with the English guidelines was related to assisting adolescents. Specifically, several items about discussing actions together with the adolescent were rejected, possibly because of a perceived need to take a more directive approach with young people. Similarly, experts rejected the suggestion of involving the family only if the adolescent agrees, reinforcing the notion of acting with limited levels of adolescent involvement. This could be linked to certain cultural characteristics in Latin America, specifically related to the aforementioned concept of *Familism*, which emphasize forms of interaction where parents play a prominent role and have significant influence, particularly in support situations [45, 93].

The focus on the role of the first aider in facilitating subsequent help-seeking by the persons is another noteworthy difference. Experts endorsed items about the need to be aware of potential obstacles that the person may encounter in seeking help, particularly within healthcare services, advising that the first aider accompany them throughout their care trajectory. This is relevant given the access and availability challenges [94] and acceptability gaps (such as fear of stigma) observed in Latin American countries [95].

Finally, it is relevant to note the consideration given by experts to vulnerable groups, highlighting special attention to groups that may be at increased risk of suicide, such as ethnic minorities or migrants. In this regard, the inclusion of an entirely new section of recommendations for older people is particularly significant, extending the life course approach of the English guidelines, which included a section on adolescents.

Regarding older adults, the experts considered it relevant for the assistant to be aware of certain indicators that may suggest a higher risk of suicide among older adults. Elderly people employ more lethal methods compared to other age groups, in addition to utilizing discreet passive means that are challenging to identify, such as ceasing nourishment or disregarding critical medical directives [96]. The experts recommended that the assistant should carefully observe any signs of the older adult becoming more isolated in the last six months. They also advised being vigilant for any increase in feelings of hopelessness in the older adult, while also paying attention to sudden decreases in autonomy and reduced participation in social interactions.

Receiving distressing medical diagnoses, either personally or from close relatives, can also be an important indicator of potential suicide risk for the experts. Furthermore, they considered that it is crucial for the assistant to be alert to any indications that the older adult may be saying goodbye to loved ones, without any apparent reason for doing so. Gifting away possessions that are perceived as most valuable may also be a sign of heightened suicide risk.

Understanding these signs can make a significant difference in offering appropriate support and intervention to those who may be at risk of suicidal thoughts or behavior among the elderly population.

### Comparison with the suicide risk guidelines adapted for other countries

China [81], Sri Lanka [82] and Brazil [83] have recently gone through a similar process of adaptation of the English-speaking guidelines during a similar time frame, allowing for cultural comparisons, although the original English items presented to the experts in Round 1 were not identical. This study showed that in the endorsement rate in Latin American countries was lower than those in China and Sri Lanka, suggesting that cultural adaptation may have been more imperative.

Importantly, both Brazil and our sites (Argentina and Chile) rejected using direct questions to ask about suicide plans (which was endorsed in China and Sri Lanka). However, it was accepted in both the former countries that “The first aider should be aware that if a person is suicidal, asking them about suicidal thoughts will not increase the risk that they will act on these”. This may be due to a cultural reluctance in Latin America to be direct when addressing other people in need [97–100]. In addition, involving the police when the suicidal person has a weapon was not accepted, as in Brazil, which may point to the police in the region being perceived as aggressive, dangerous, and corrupt, rather than as helpers to citizens in crisis. However, in Argentina and Chile it was accepted that the police should be summoned only after not being able to contact a family member of the person or someone close to them and trying to get the person to hand over the weapon without confrontation.

Argentina and Chile were the only sites to reject the statement about the first aider avoiding the use of terms to describe suicide that promote stigmatizing attitudes, e.g. ‘commit suicide’ or refer to a suicide attempt as having ‘failed’ or been ‘unsuccessful’. Health professionals had an even lower endorsement rate for this statement compared to the lived experience panel which may indicate the presence of significant but underestimated stigmatizing attitudes among mental health professionals towards individuals with mental health problems [101].

### Comparison with other MHFA guidelines adapted for Chile and Argentina

As with the adaptation of the English-speaking guidelines for problem drinking [84] and depression [85], the adaptation of the suicide risk guidelines showed that experts endorse an indirect, warm and non-confrontational approach to establish a helping relationship. Another common element was the consideration of social determinants of health, as manifested in recommendations about vulnerable groups or exposure to risks, such as economic stress or belonging to a marginalized group (LGBTIQ, ethnic minorities, older adults). Another common theme was that the first aider should not share their own feelings or thoughts, or to adopt an “I-statement” position in the process of providing assistance. This is likely aimed at avoiding trivializing the help-seeker’s story or the bias of interpreting someone else’s problems based on one’s own experiences. There is also a tendency among Chilean and Argentinean experts to disapprove of seeking self-help information, possibly due to considering it as low-quality information that may not be helpful. Across all guidelines, health professionals were more likely to consider that the first aider should take the lead in the helping actions rather than engaging in collaborative decision-making with the person receiving help, possibly indicating a lower value placed on the person’s autonomy compared to that seen in other countries, tendency probably related to the stigma attributed to the person at risk of suicide [102].

### Strengths and limitations

Following a similar methodology to previous studies, the cultural adaptation of English Mental Health First Aid guidelines to the Chilean and Argentinian context [84, 85] drew on a wide range of expertise. Giving equal weight to the views of health professionals and people with lived experience aligns with strong recommendations of those leading mental health research [102].

However, participants from the professional panel were mainly from Chilean and Argentinian metropolitan areas while participants from the lived experience panel were only from Chile and did not specify their location. This limitation may have affected the comparability of panels and the generalizability of results. Additionally, a methodological challenge is the lack of recommendations from individuals who have died by suicide. In this design, the inclusion of family members and caregivers aimed to address this difficulty through gaining insights from people who have lost someone to suicide. However, our study design and sample size precludes comparisons between the two groups. Future research could involve surviving family members to further enhance the understanding of suicidal behavior.

Finally, it is imperative to acknowledge that, although the Delphi methodology is most suitable for gathering evidence on actions that are difficult to experimentally test, any interventions based on the culturally adapted guidelines should be rigorously tested, including through pilot testing to explore the potential for harms and to suggest improvements, and subsequently through randomised controlled trials. Further consideration should also be given to how MHFA training might be implemented in the health and education systems in Chile and Argentina, including who might train as MHFA Instructors and how to ensure sustainable funding for training, for example with Ministry of Health, philanthropic or private sector funding, all of which have happened in other countries. It is critical to consider how these guidelines and the training that they inform can be implemented into practice in primary healthcare, educational institutions, and community environments. This will vary according to country context, e.g. for over three decades, Chile has been developing a model of mental healthcare centered on primary health care, where more than 80% of these services are provided [103]. The current National Suicide Prevention Program [58] has focused its efforts on schools and community settings, in which the findings of this research and subsequent research endeavors are likely to be highly relevant. Other emerging initiatives aimed at building community capacity through the coordination of health programs also have sufficient proximity to forge alliances that can leverage the implementation of these guidelines and related training [74, 104]. In Argentina, the National Suicide Prevention law [60] and the Comprehensive Care Program for the Problem of Suicide [105] have both prioritised strengthening the response capacity of local healthcare and psychosocial support networks; however, an integrated and comprehensive training program for community members is still lacking. The mental health first aid guidelines for individuals at suicide risk developed in this study, as well as the training that they will inform, may assist in improving suicide prevention efforts in different community settings (including schools, universities, work, and public places) through provision of specific implementation detail.

## Conclusion

A Delphi expert consensus study involving health professionals and people with lived experience was used to adapt the mental health first aid guidelines for a person at risk of suicide in Chile and Argentina. Items from the English-language guidelines related to asking directly about suicide risk were not endorsed, along with some items about respecting the autonomy of the person, particularly in the case of adolescents. An additional section on suicide risk in older People was a notable addition. Further research should address uptake of the guidelines in Chile and Argentina is necessary and incorporation into MHFA training for these countries.

### Electronic supplementary material

Below is the link to the electronic supplementary material.

Supplementary Material 1: Statements that were presented to the panels and their rating across 2 rounds of the study.

Supplementary Material 2: Expert consensus Spanish guidelines for helping a person who is suicidal risk.

## Data Availability

The data supporting our findings is attached as Additional file 1, which contains all the statements that were presented to the panels and their endorsement rates.

## List of abbreviations

MHFA: Mental Health First Aid
LMICs: Low- and middle-income countries
HICs: High Income Countries
