# Supplementary material for: Development of mental health first-aid guidelines for suicide risk: a Delphi expert consensus study in Argentina and Chile
Source: BMC Psychiatry. 2023 Dec 11;23:928. doi: 10.1186/s12888-023-05417-0 (PMC10712185; doi:10.1186/s12888-023-05417-0)
Supplement: Supplementary file 1 — Supplementary Material 1: Statements that were presented to the panels and their rating across 2 rounds of the study. [file 12888_2023_5417_MOESM1_ESM.pdf]

CÓMO BRINDAR AYUDA A UNA PERSONA EN RIESGO DE SUICIDIO

GUÍA DE PRIMEROS AUXILIOS EN SALUD MENTAL

El suicidio se puede prevenir. La mayoría de las personas en riesgo de suicidio no quieren morir. Simplemente no quieren vivir con el dolor que experimentan. Hablar abiertamente sobre pensamientos y sentimientos suicidas puede salvar una vida. No subestimes tus habilidades para ayudar a una persona en esta situación, incluso para salvarle la vida.

En esta guía encontrarás un conjunto de recomendaciones para ayudar a alguien en riesgo de suicidio divididas en diferentes secciones:
- La identificación del riesgo de suicidio.
- La evaluación de la gravedad del riesgo.
- Qué y cómo hablar con alguien en riesgo de suicidio.
- La elaboración de un plan de seguridad.
- Aspectos para poblaciones específicas, etc.

Al finalizar podrás ver detalles de cómo se realizó esta guía y sugerencias para su utilización o consultas adicionales.

La identificación del riesgo de suicidio

Es importante que seas capaz de reconocer las señales de advertencia y actuar rápidamente si crees que alguien está considerando suicidarse. Si la persona en riesgo de suicidio ya se ha dañado a sí misma, debes administrar primeros auxilios y llamar a los servicios de emergencia, solicitando una ambulancia.

No debes suponer que la persona mejorará sin ayuda ni que la persona buscará ayuda por sí misma. También debes ser consciente de que no en todos los casos que una persona está pensando seriamente en el suicidio lo va a decir, e incluso podría mostrarse calmada.

Es importante que sepas que si una persona no tiene ideas suicidas, preguntarle no va a poner esta idea en su cabeza si no estaba con anterioridad, ni aumentará el riesgo de que actúe en base a ellas ni inducirá al suicidio. Al contrario, le dará la oportunidad de hablar sobre sus problemas y de hacerle saber que a alguien le importa, ayudando a disminuir el riesgo y ofrecer una alternativa para buscar una mejor solución y más efectiva al problema que le genera sufrimiento. Al mismo tiempo, nunca debes sugerir ideas, pensamientos o formas de quitarse la vida a la persona en riesgo de suicidio.

Permite que la persona en riesgo de suicidio hable abiertamente de sus sentimientos. Una persona en riesgo de suicidio puede sentir alivio al hablar. Si las circunstancias lo permiten, procura encontrar un momento y lugar adecuado, que inspire confianza y seguridad, para conversar con la persona. Si la persona en riesgo de suicidio dice que está escuchando voces, debes preguntarle qué le dicen las voces (ya que las alucinaciones acustico-verbales podrían ser relevantes para sus ideas de suicidio actuales) y buscar ayuda especializada.

Si te sientes incapaz de preguntarle a la persona si está pensando en el suicidio, si sientes que no está funcionando tu intento de ayuda, si no puedes

establecer una conexión con la persona, si sientes que la persona no quiere hablar contigo o si te sientes abrumado o incapaz de contener o escuchar la gravedad de lo que cuenta la persona, debes ofrecerle encontrar a alguien más con quien la persona pueda hablar y buscar ayuda en un servicio de salud inmediatamente.

Es importante comprender que la persona puede no querer hablar contigo, por lo que ayudarla a encontrar otra persona con quien hablar puede ser de mucha ayuda; a veces la persona puede estar más permeable a hablar con alguien de la misma etnia, edad, sexo o género. En cualquier caso, es más importante querer realmente ayudar que ser de la misma edad, género o cultura de la persona.

Se consciente de tus propias ideas acerca del suicidio y el impacto de estas ideas sobre tu capacidad para proporcionar asistencia (por ejemplo, creencias de que el suicidio es incorrecto o que es una opción racional). Asimismo, es importante que utilices un lenguaje apropiado cuando te refieras al suicidio usando los términos "suicidio" o "muerte por suicidio", teniendo en cuenta que las diferentes culturas tienen diferentes creencias y actitudes sobre el suicidio. Algunas personas encuentran en la explicación religiosa o en la idea del pecado algo que sirve de freno para el suicidio, en cuyo caso debes ser cuidadoso de no hacer comentarios que cuestionen este freno.

Es común que sientas gran inquietud o que te veas afectado cuando alguien revela ideas de suicidio. Sin embargo, es importante que intentes mostrarte tranquilo y confiado frente a la crisis suicida, ya que puede tener un efecto tranquilizador para la persona. Ahora bien, ante un riesgo elevado de suicidio, actúa con premura para activar la red de apoyo y/o los servicios especializados apropiados, evaluando lo más pronto posible los eventuales integrantes de una red de sostén para la persona en esta circunstancia. Evita expresar reacciones negativas a las ideas de suicidio (por ejemplo, juzgar negativamente a la persona, entrar en pánico, manifestar enojo o conmoción, respondiendo con calma y empatía). En el plano no verbal, es importante que te entrenes en utilizar tu cuerpo y entonaciones de voz de modo de generar mayor confianza en la persona.

Evaluación de la gravedad del riesgo de suicidio

Toma en serio todos las ideas o expresiones de suicidio, actuando en base a éstas sin tomarlas como "llamado de atención" o "manipulación"; más bien puede ser una forma de pedir de ayuda. La falta de un plan de suicidio no es suficiente para garantizar la seguridad. La urgencia de tomar medidas debe estar determinada por el reconocimiento de señales de advertencia de suicidio. Recuerda que debes intentar identificar a alguien de la red de la persona que sepa de la situación y pueda brindar apoyo.

En caso que sepas o creas que la persona en riesgo suicida tiene un plan, averigua si ya ha tomado medidas o dispuso los pasos para concretar su plan para quitarse la vida. Esto es importante ya que quien tiene mayor riesgo de

actuar en el futuro cercano en base a sus ideas de suicidio es quien tiene un plan de suicidio específico, tomó medidas, dispuso cuáles serían los pasos para llevarlo a cabo y pensó en un momento específico para hacerlo.

Dado que el consumo de alcohol y/o drogas es un factor facilitador para la consecución de un intento de suicidio, debes permanecer con la persona si detectas que está bajo el efecto de esta o alguna otra sustancia, y reevaluar la situación una vez pasado el cuadro de intoxicación.

Pregúntale a la persona cómo se siente en ese momento y si le ha contado a alguien cómo se siente. Para determinar el riesgo de suicidio, algunas preguntas importantes que debes hacer son: (a) si ha estado usando drogas o alcohol: ya que esto puede ser un factor facilitador para la consecución de un intento de suicidio, (b) Si ha recibido o recibe tratamiento por salud mental, por ejemplo, medicación, (c) si alguna vez ha realizado un plan para quitarse la vida en el pasado, (d) si alguna vez intentó quitarse la vida en el pasado, (e) si hubo cambios o situaciones actuales contextuales estresantes que puedan orientarte a pensar en una mayor o menor gravedad de la situación de la persona y, (f) si existen personas de apoyo actuales en la vida de la persona en riesgo.

Es importante que consideres el grado de impulsividad de la persona y capacitarte para evaluarlo, ya que la impulsividad podría ser un factor precipitante del suicidio aun sin un plan para realizarlo.

¿Qué es la impulsividad?

La impulsividad es una predisposición a tener reacciones rápidas e imprevistas a estímulos internos o externos sin tener en cuenta las consecuencias negativas de estas reacciones para el individuo impulsivo[1]; es decir, no existe premeditación del comportamiento.

¿Cómo puedes darte cuenta de que una persona demuestra impulsividad?

Que la persona responda sin pensar, que se siente inquieta mientras uno habla por periodos largos de tiempo, o que se muestra impaciente son algunos de los elementos que pueden ayudar a identificar si la persona tiene rasgos de impulsividad[2].

Por otro lado, debes capacitarte en evaluar niveles de alteración y/o agitación para determinar la urgencia en la búsqueda de ayuda profesional.

Como complemento, puede resultarte de utilidad conocer la siguiente pauta para la evaluación y manejo de riesgo elaborada en base a la Escala de Severidad Suicida de Columbia (C-SSRS)[3]. Las respuestas de "SÍ" en cuadros amarillos corresponden a riesgo bajo, color anaranjado a riesgo moderado, y color rojo a riesgo alto.

Referencias:

[1] Moeller FG, Barratt ES, Dougherty DM, Schmitz JM, Swann AC. Psychiatric aspects of impulsivity. Am J Psychiatry. 2001 Nov;158(11):1783-93.

[2] Mann JJ, Waternaux C, Haas GL, Malone KM. Toward a clinical model of suicidal behavior in psychiatric patients. Am J Psychiatry. 1999 Feb;156(2):181-9.

[3] Posner, K. (2008). Escala de Severidad Suicida de Columbia (C-SSRS). Versión para Chile – Pesquisa con puntos para Triage. Kelly Posner, Ph.D © 2008. Revisada para Chile por Vania Martínez 2017.Tomada de: Ministerio de Salud de Chile (2019). Recomendaciones para la prevención de la conducta suicida en establecimientos escolares. Programa Nacional de Prevención del Suicio, Chile.

MENTAL HEALTH FIRST AID
Chile - Argentina

CÓMO BRINDAR AYUDA A UNA PERSONA EN RIESGO DE SUICIDIO

GUÍA DE PRIMEROS AUXILIOS EN SALUD MENTAL

**Formule las preguntas 1 y 2**	**Último mes**	
	**SI**	**NO**
**1) ¿Has deseado estar muerto (a) o poder dormirte y no despertar?**<br>La persona confirma que ha tenido ideas relacionadas con el deseo de estar muerta o de no seguir viviendo, o el deseo de quedarse dormida y no despertar.		
**2) ¿Has tenido realmente la idea de suicidarte?**<br>Pensamientos generales y no específicos relativos al deseo de poner fin a su vida / suicidarse (por ejemplo, “He pensado en suicidarme”) sin ideas sobre cómo quitarse la vida (métodos relacionados, intención o plan).		
**Si la respuesta es “Si” a la pregunta 2, formule las preguntas 3, 4, 5, y 6.**<br>**Si la respuesta es “No” continúe a la pregunta 6.**		
**3) ¿Has pensado en cómo llevarías esto a cabo?**<br>El o la estudiante confirma que ha tenido ideas suicidas y ha pensado en al menos un método. Esto se diferencia de un plan específico con detalles elaborados de hora, lugar o método, pues en este caso existe la idea de un método para matarse, pero sin un plan específico. Incluye también respuestas del tipo: “He tenido la idea de tomar una sobredosis, pero nunca he hecho un plan específico sobre el momento, el lugar o cómo lo haría realmente...y nunca lo haría”.		
**4) ¿Has tenido estas ideas y en cierto grado la intención de llevarlas a cabo?**<br>Se presentan ideas activas de quitarse la vida y el o la estudiante refiere que ha tenido cierta intención de llevar a cabo tales ideas, a diferencia de ideas suicidas sin intención en respuestas como “Tengo los pensamientos, pero definitivamente no haré nada al respecto”.		
**5) ¿Has comenzado a elaborar o has elaborado los detalles sobre cómo suicidarte? ¿Tienes intenciones de llevar a cabo este plan?**<br>Se presentan ideas de quitarse la vida con detalles del plan parcial o totalmente elaborados, y el o la estudiante tiene cierta intención de llevar a cabo este plan.		
**Siempre realice la pregunta 6**		
**6) ¿Alguna vez has hecho algo, comenzado a hacer algo o te has preparado para hacer algo para terminar con tu vida?**<br>Pregunta de conducta suicida: Ejemplo: ¿Has juntado medicamentos / remedios, obtenido un arma, regalado cosas de valor, escribir un testamento o carta de suicidio?, ¿has sacado remedios del frasco o caja, pero no las has tragado, agarrado un arma pero has cambiado de idea de usarla o alguien te la ha quitado de tus manos, subido al techo pero no has saltado al vacío?; o ¿realmente has tomado remedios, has tratado de dispararte con un arma, te has cortado con intención suicida, has tratado de colgarte, etc.?<br><br>Si la respuesta es “Si”. ¿Fue dentro de los últimos 3 meses?	**Alguna vez en la vida**	
	**En los últimos 3 meses**	

CÓMO BRINDAR AYUDA A UNA PERSONA EN RIESGO DE SUICIDIO

GUÍA DE PRIMEROS AUXILIOS EN SALUD MENTAL

La asistencia inicial

No debes ponerte en peligro mientras ofreces apoyo a la persona en riesgo de suicidio. Para garantizar la seguridad de la persona, trabaja en colaboración con ella en lugar de actuar solo/a. Es importante que no dejes solo a alguien en riesgo inminente de suicidio, aún cuando la persona rechace ser ayudada. Considera que aún cuando la persona se comprometa a ciertos acuerdos, esto no es garantía de que los vaya a cumplir.

Si sospechas que hay un riesgo inminente de que la persona lleve a cabo sus ideas de suicidio, debes actuar rápidamente, incluso si no estás seguro. Prepárate para que la persona posiblemente exprese enojo y se sienta traicionado/a debido a tu intento de evitar su suicidio o al ayudarla a obtener ayuda profesional.

A continuación se presentan recomendaciones de acciones de ayuda dependiendo de diferentes situaciones de riesgo:

Recomendación para situaciones comunes

Si la persona...	Dialoga con la persona las acciones de ayuda	Aliéntala a buscar ayuda profesional lo antes posible (consultar a un profesional de salud mental o en un servicio de salud mental)	Busca el permiso de la persona para contactar a su médico habitual o profesional de salud mental en el caso en que tuviera uno	Llama a un centro de salud, una línea telefónica para crisis de salud mental o línea directa de prevención de suicidio para pedir asesoramiento
Está en cualquier nivel de riesgo	✓	✓		
No puede comprometerse a no hacerse daño	✓	✓		
Tiene un plan específico	✓	✓	✓	
Tiene los medios para llevar a cabo el plan de suicidio	✓	✓	✓	✓

No está de acuerdo en entregar los medios que pensaba utilizar para su plan suicida	✓	✓	✓	✓
Ha intentado suicidarse en el pasado	✓	✓	✓	
Se niega a buscar ayuda o rechaza la ayuda profesional				✓
No quiere hablar con alguien cara a cara			✓	✓

Si la persona está en situación confrontativa con tus sugerencias, debes privilegiar la empatía y el acompañamiento por sobre la persuasión. Si tiene un arma, debes contactar a un familiar de la persona o alguien cercano a ella, tratar que entregue el arma sin llegar a confrontar y avisar a la policía si eso no fuese posible. Si debes llamar a la policía, infórmales que la persona está en riesgo de suicidio, con el fin de ayudarles a responder de manera apropiada.

Es importante que busques información sobre los recursos y servicios disponibles de ayuda para una persona que está considerando suicidarse, de modo de proporcionar esta información cuando sea necesario. Conversa con la persona acerca de cómo ponerse en contacto con un centro de Salud Mental (dónde hallar un centro cercano, cómo conseguir un turno, qué programas existen para poder ayudarla, etc.). Si es necesario que tú tomes contacto con un profesional de la salud para hablar acerca de la persona, es preferible hacerlo con un profesional que la persona ya conozca y confíe.

Además de brindarle información de lugares disponibles, considera acompañar presencialmente a la persona en la búsqueda de ayuda a un centro de salud si nadie de la familia o cercano a la persona está disponible. Considera que la persona puede encontrarse con obstáculos en el acceso a los servicios de salud en relación con el riesgo de suicidio, por lo que debes acompañarla para superarlos.

Es importante que estés consciente de que quizás no tengas éxito en la prevención del suicidio. Ten siempre presente tu auto-cuidado después de ayudar a alguien que está en riesgo de suicidio.

En Chile, la línea *4141 es un teléfono de ayuda gratuito para la prevención del suicidio que funciona todos los días las 24 horas, con profesionales psicólogos/as capacitados/as para apoyar a personas que presentan dolor emocional extremadamente intenso, con ideas de hacerse daño o querer morir.

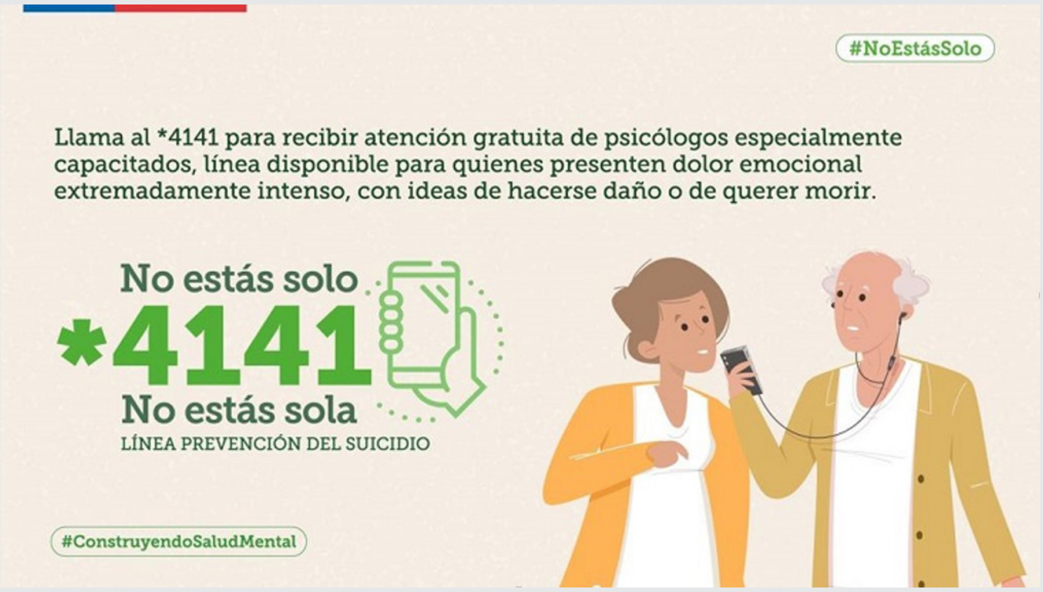

En Argentina tenemos algunas opciones de asistencia gratuita y telefónica de acuerdo en qué región del país te encuentres:

Desde todo el país:
Dispositivo de Orientación y Apoyo en la Urgencia de Salud Mental: **0800-999-0091**
(Atención las 24 hs, todos los días).

Si te encontrás en la Ciudad Autónoma de Buenos Aires (CABA):
Salud Mental Responde: **0800-333-1665**
(Atención las 24 hs, todos los días).

Si te encontrás en la Provincia de Buenos Aires:
Subsecretaría de Salud Mental, consumos problemáticos y violencias en el ámbito de la salud: **0800-222-5462**

Línea de prevención del suicidio (Centro de Asistencia al Suicida; CAS):
135 (línea gratuita) o **(011)5275-1135 o 0800-345-1435** desde todo el país.

Qué y cómo hablar con una persona que presenta riesgo de suicidio

Resulta esencial que procures encontrar un espacio tranquilizador o que provea confort a la persona para mantener este tipo de conversación; debemos evitar la conversación "de pasillo". El primer contacto (incluyendo el lenguaje verbal y no verbal, y el contexto) puede ser crítico para crear un vínculo que posibilite la ayuda.

Cuando te encuentres hablando con una persona con ideas o riesgo suicida, debes comunicarle a la persona en riesgo que te preocupas por ella y que quieres ayudarla, de forma tranquila hacerle saber que quieres escuchar lo que la persona tenga para decir. Debes prestar toda tu atención y mostrarte paciente cuando la persona expresa sus sentimientos. Es importante que estés atento a tu lenguaje corporal, asegurando que no comuniques falta de interés o alguna actitud negativa a lo que la persona expresa. Debes buscar aclarar los puntos importantes con la persona para asegurarte de que estás entendiendo completamente la situación. Resumir en voz alta lo que la persona ha manifestado es una demostración de que estás escuchando atentamente. Esto puede acercar a la persona a hablar con mayor confianza.

Preguntar de forma abierta qué está pensando y/o sintiendo la persona es importante, así como cuáles son los problemas que están detrás de esta crisis, de forma empática y brindando apoyo y comprensión a cualquier respuesta de la persona. Es fundamental en este punto no emitir juicios de valor ("está bien", "está mal", "esto no es así") frente a las respuestas de la persona, y validar los sentimientos o emociones ("entiendo que estés pasando por un momento muy difícil", "te noto muy abrumado por todo esto"). Muchas veces, que las personas expresen sus pensamientos y/o sentimientos (por ejemplo, llorar, expresar enojo o gritar) es una forma de pedido de ayuda y, en muchas otras, pensar en matarse es un intento de escapar a los problemas o al sufrimiento, por lo que debes permitirle a la persona expresarse. Debes enfatizar en que puede ser difícil hablar de ellos, y a su vez, que es la mejor oportunidad para poder hablar de cosas que son dolorosas.

Debes permitirle a la persona hablar respecto a sus razones para morir, sólo si es que desea expresarlo. Si bien debes orientarte a una conversación basada en la comprensión y la empatía, en algunos momentos debes adoptar una actitud más directiva y considerar cuestionar algunos pensamientos (por ejemplo, desafiar la idea de que el suicidio soluciona los problemas) y evitar validar acciones suicidas (para no cometer errores puede servir validar emociones, y no pensamientos o acciones). En este punto, es esencial evaluar si existen elementos en su contexto que pueden poner a la persona en riesgo (por ejemplo, algún medio letal disponible) y a su vez, reforzar las cosas que la mantienen con vida (o la resguardan del riesgo).

Es importante que tengas en cuenta la pertenencia a un grupo minoritario, como inmigrantes, pueblos originarios o comunidad transgénero, que podrían aumentar las probabilidades de riesgo de suicidio de la persona.

Qué NO hacer al hablar con una persona con riesgo de suicidio

Saber qué no decir y qué no hacer es tan importante, o más, que saber que sí puedes hacer, cuando se conversa con alguien con ideas de suicidio. A pesar de ello una recomendación importante es no dejar que el miedo que puedas sentir de decir las palabras equivocadas o de no decir las palabras más adecuadas termine por evitar que la persona en riesgo de suicidio pueda hablar, que es el objetivo principal de la conversación.

Si queremos que la conversación sea empática y efectiva, no debes discutir o debatir con la persona sobre sus pensamientos de suicidio, o sobre si el suicidio es correcto o incorrecto (juicios de valor). Tampoco debes minimizar los problemas que tiene la persona con riesgo suicida (por ejemplo, diciendo "no es tan grave"). Los problemas de una persona son tan válidos como los de cualquier otra persona. Es preferible evitar frases tales como "no te preocupes", "anímate", "tienes todo a tu favor", "todo estará bien" o "la vida es bella". Por otro lado, no intentes demostrar que la persona en riesgo de suicidio no tiene intención de quitarse la vida, o desafiarla a que lo demuestre o intente, o decirle que "simplemente lo haga".

Los trastornos mentales no son necesarios ni suficientes para tener ideas de suicidio. Por lo tanto, no es adecuado intentar dar a la persona en riesgo de suicidio un diagnóstico de un problema de salud mental o enfermedad psiquiátrica. Primero, porque en muchas ocasiones el problema de salud mental no es la causa de la idea suicida; y segundo, un gran porcentaje de personas que mueren por suicidio, no tienen un trastorno mental. Es igual de importante no asumir que si la persona ha tenido intentos de suicidio con anterioridad, la probabilidad de morir por suicidio es menor. Al contrario, es un factor de riesgo adicional.

Debes evitar usar amenazas o buscar el modo de culpabilizar a la persona para evitar el suicidio (por ejemplo, diciéndole que irá al infierno o que arruinará la vida de otras personas si se mata).

Por último, no debes tomar a título personal cualquier acción o palabra dañina en las que pueda haber incurrido la persona en riesgo de suicidio. Una forma compasiva de acercamiento es pensar que las palabras o acciones de una persona con riesgo de suicidio seguramente están influidas por un intenso dolor emocional.

El efecto que esta conversación te puede provocar

Es esperable que las relaciones interpersonales tengan efectos en todos los que participan de las mismas. La conversación con alguien con riesgo de suicidio no es la excepción. Debes aceptar y tener presente que podrías experimentar diferentes emociones en el proceso de ayudar a la persona (cansancio, enojo, frustración, culpa), y que eso es razonable. Considera tener apoyo psicológico para ti mismo a propósito de la tarea de dar apoyo a personas en riesgo de suicidio. Esto podría reducir el agotamiento y facilitar que estés en condiciones para ayudar a una próxima persona con este riesgo.

La elaboración de un acuerdo para prevenir el suicidio: el plan de seguridad.

Uno de los objetivos de la conversación con alguien con ideas de suicidio es elaborar en forma colaborativa un plan para utilizar en momentos de crisis; esta estrategia suele llamarse “plan de seguridad”. Es colaborativa porque –en la medida de lo posible– debes involucrar a la persona en riesgo de suicidio en las decisiones sobre este plan.

El plan de seguridad debe ser claro, describiendo qué se hará, quién lo hará y cuándo se llevará a cabo, y debe centrarse en lo que la persona en riesgo de suicidio debe hacer, y no en lo que no debería hacer. Debes pedirle a la persona en riesgo de suicidio que proporcione una lista de contactos de personas que la puedan ayudar en esos momentos, y acordar a quién llamará cuando se sienta en riesgo de suicidio. Los contactos del médico o profesional de salud mental de la persona en riesgo de suicidio, una línea de ayuda para personas en riesgo de suicidio o una línea de atención en crisis las 24 horas, así como amigos y familiares que lo ayudarán en una emergencia, son contactos telefónicos necesarios. Esto resullta de fundamental importancia en el caso de que la persona se sienta incapaz de continuar con el acuerdo de no intentar el suicidio.

Debes tener en cuenta que el plan de seguridad debe durar un tiempo que sea posible de cumplir por la persona en riesgo de suicidio, de modo que pueda sentirse capaz de alcanzar el acuerdo, brindándole la oportunidad de sentir que pudo lograr algún avance. Finalmente, es importante remarcar que los planes de seguridad pueden requerir ajustes ya que la situación puede modificarse de un día a otro, a lo largo del tiempo.

Componentes del plan de seguridad (para realizarlo en formato escrito con una copia para la persona en riesgo y otra para el asistente que lleva a cabo la entrevista)[4]:

1. Identificar y enumerar señales de alarma (emociones, pensamientos, sensaciones corporales, situaciones del entorno) que nos avisan que la persona está entrando o ya se encuentra en crisis suicida.

2. Estrategias de afrontamiento: qué puede hacer la persona con riesgo de suicidio para distraerse, pero sin depender de terceros (focalizar en cosas que haya hecho efectivamente la persona en anteriores crisis que no terminaron en intento de suicidio).

3. Llamado a otras personas de confianza con el objetivo de distraerse del malestar.

4. Llamado a personas de confianza con riesgo con el objetivo de que funcionen de ayuda o soporte en la crisis suicida.

5. Contacto con profesionales de la salud mental: puede ser su psicoterapeuta, su psiquiatra o incluso teléfonos de emergencia con las que la personas pueda contactar para pedir ayuda. Cuantas más personas se puedan contactar, mejor aún.

6. Disminuir medios letales disponibles: procurar acordar con la persona en riesgo para deshacerse de medios potencialmente letales (ej. pastillas que piensa tomar en un intento de suicidio).

[4] Adaptado de Stanley B, Brown GK. Safety planning intervention: a brief intervention to mitigate suicide risk. Cognit Behav Pract. 2012;19(2):256-264.

CÓMO BRINDAR AYUDA A UNA PERSONA EN RIESGO DE SUICIDIO

GUÍA DE PRIMEROS AUXILIOS EN SALUD MENTAL

Cosas que deberías saber

Existen algunas premisas que debes saber para establecer una conversación con una persona con riesgo de suicidio. Por supuesto, es importante conocer cuáles son los factores de riesgo que aumentan la probabilidad de que un suicidio suceda. Sin embargo, no es lo único a tener en cuenta. Existen razones por las cuales las personas tienen ideas de suicidio, y sobre todo, razones por las que estas personas no piden ayuda. A continuación, se enumeran los hechos que son imprescindibles que sepas:

- Cualquier persona puede tener pensamientos de suicidio.

- El suicidio se puede prevenir.

- Hablar abiertamente sobre pensamientos y sentimientos suicidas puede salvar una vida.

- Hablar de suicidio no "meterá la idea" en la cabeza de alguien que no pensaba quitarse la vida.

- A menos que alguien te lo diga, la única forma de saber si una persona está pensando en suicidarse es preguntándole.

- La mayoría de las personas en riesgo de suicidio no quieren morir; simplemente no quieren vivir con el dolor.

- Es más importante hablar genuinamente que evitar hablar por miedo a equivocarse o a no decir "todas las cosas correctas".

- Existe relación entre el suicidio y la enfermedad mental, pero no siempre las conductas suicidas son precedidas por un diagnóstico de enfermedad mental.

- El uso de alcohol u otras drogas puede aumentar el riesgo de que una persona lleve a cabo sus pensamientos suicidas.

- No deberías subestimar tus propias habilidades para ayudar a una persona en riesgo de suicidio, incluso para salvarle la vida.

- A pesar de que puedes ofrecer apoyo, no eres responsable de las acciones o el comportamiento de otra persona, y no puedes controlar lo que ésta haga.

Por último, resulta esencial que conozcas cuáles son los servicios locales que pueden ayudar a personas en riesgo de suicidio (es decir, hospitales, centros de salud mental, equipos de crisis de alcance móvil, líneas de asistencia para la prevención del suicidio, servicios de emergencia locales, entre otros).

La confidencialidad

La conversación con alguien con riesgo de suicidio puede ser una oportunidad única; sin embargo, existen algunos puntos relevantes respecto a la confidencialidad de esta conversación.

Como asistente de primeros auxilios nunca debes aceptar mantener en secreto el riesgo de suicidio, como tampoco debes flexibilizar y aceptar mantener en secreto los planes suicidas que tenga la persona. En este sentido, una conducta recomendada es tratar con respeto a la persona en riesgo de suicidio e involucrarla en las decisiones sobre qué otra persona debe saber acerca de la crisis suicida. De lo contrario, si la persona en riesgo de suicidio se niega a dar permiso para divulgar información sobre sus pensamientos suicidas, puede que necesites romper esa confidencialidad para garantizar su seguridad. En caso de que esto ocurra, debes ser transparente y comunicarle a la persona a quién se notificará (familiar, amigo, policía, servicios de emergencia, etc).

Es preferible que la persona se enoje por compartir sus pensamientos suicidas sin su permiso (para obtener ayuda) que permitir que la persona se quite la vida.

Consideraciones para poblaciones especiales: Adolescentes

Cuando detectes a un adolescente con riesgo de suicidio es clave no dejarlo/a solo/a. Sé cuidadoso de no trivializar o restar importancia a los temas que el/la adolescente pueda esgrimir en relación a sus ideas de suicidio, aún si parecen de una magnitud diferente a los de personas adultas. Con el objetivo de mantener una conversación empática y de cercanía, es recomendable que hagas un esfuerzo adicional para utilizar un lenguaje más afín al adolescente y menos técnico para poder establecer una mejor conexión.

De la misma forma que con adultos, debes tratar al adolescente con respeto e involucrarlo en las decisiones sobre quién más podrá o deberá estar al tanto de la crisis suicida, ya que, siempre que sea posible y preferentemente con el

acuerdo del/la adolescente, es prioridad contactar o involucrar a la familia y/o personas adultas responsable de apoyo del/la adolescente que está en riesgo de suicidio.

Recomendación par guiar la conversación y asistir a adolescentes con riesgo de suicidio

**Si el adolescente en riesgo de suicidio**	**Discute con el adolescente qué acciones debe tomar para obtener ayuda**	**Alienta al adolescente a obtener la ayuda profesional adecuada lo antes posible (consultar a un profesional de salud mental o a alguien en un servicio de salud mental)**	**Busca el permiso del adolescente para contactar a su médico habitual o profesional de salud mental en el caso en que tuviera uno**	**Llama a un centro de salud, una línea telefónica para crisis de salud mental o línea directa de prevención de suicidio para pedir asesoramiento**	**Sigue alentando al adolescente a consultar a un profesional de salud mental.**	**Insiste para que se ponga en contacto con una línea directa de prevención de suicidio para obtener orientación sobre cómo recibir ayuda.**
Tiene tendencias suicidas (ideas/ intención suicida actuales) o ha intentado suicidarse en el pasado *	✓	✓				
Tiene un plan específico		✓	✓			
Tiene los medios para llevar a cabo su plan suicida o no acepta darte las cosas que pretende usar para suicidarse o no puede comprometerse a mantenerse a salvo		✓	✓	✓		
Si el adolescente en riesgo de suicidio es reacio a buscar ayuda o rechaza la ayuda profesional				✓	✓	✓

*En caso de que haya intentado suicidarse en el pasado, puedes buscar el permiso del adolescente para contactar a su médico habitual o profesional de salud mental en el caso en que tuviera uno.

CÓMO BRINDAR AYUDA A UNA PERSONA EN RIESGO DE SUICIDIO

En caso de que no puedas persuadir al adolescente en riesgo de suicidio para que obtenga ayuda, debes orientarlo/a a obtener ayuda de un amigo de confianza, una línea de ayuda o un profesional de salud mental. Es decir, debes asegurarte que el adolescente reciba ayuda de un profesional de la salud, un grupo de apoyo o una organización comunitaria relevante.

Consideraciones para poblaciones especiales: Personas Mayores

Las personas mayores (> 65 años) suelen utilizar métodos de suicidio más letales que los utilizados por otros grupos etarios. En ocasiones, la forma de suicidarse es por medio de formas pasivas (por ejemplo, no ingerir alimentos o abandonar las indicaciones médicas).

Como asistente de primeros auxilios debes estar atento a la presencia de algunos de los siguientes indicadores de la vida de una persona adulta mayor que pueden sugerir un mayor riesgo de intentar suicidarse:

- Incremento en el aislamiento en los últimos 6 meses.

- Incremento en la desesperanza que manifiesta la persona adulta mayor.

- Disminución en la autonomía (la familia le dice que no va a poder vivir solo/a o que no va a poder manejar un vehículo si lo tenía, etc.), seguida de una importante retracción para interactuar con otros.

- Recibir un diagnóstico médico propio o de alguien muy cercano que le hubiera resultado muy perturbador (por ejemplo, que anticipa como invalidante, doloroso, o que le quitará autonomía).

- Una creciente dificultad para manejar el dolor físico.

- Dar señales de querer despedirse de gente cercana no habiendo nada que sugiera la necesidad de hacerlo.

- Se ha impuesto la tarea de regalar lo que considera sus bienes más valiosos.

- Realizar afirmaciones tales como: decir que no quiere seguir viviendo, que los demás estarían mejor sin él/ella, que quiere terminar con "todo esto", que es mejor estar muerto que vivir así, que no quiere seguir siendo una carga para los demás, etc.

Algunas recomendaciones para cuidar tu propia salud mental

Puede ser bueno hacer cosas que mejoren tu propio estado de ánimo o tu salud mental después de ayudar a otras personas. Las actividades que se sabe que son útiles para mejorar el estado de ánimo y reducir la ansiedad incluyen comer bien, mantener unos hábitos de sueño regulares, practicar técnicas de relajación (por ejemplo, la relajación muscular progresiva), mantenerse físicamente activo, hablar con personas que te apoyen, supervisar la tarea realizada con un colega más experimentado, hacer saber a otras personas cómo te sientes, programar actividades agradables (en particular, las que dan una sensación de logro) y hacer otras cosas que sabes que han sido útiles en el pasado.

CÓMO BRINDAR AYUDA A UNA PERSONA EN RIESGO DE SUICIDIO

GUÍA DE PRIMEROS AUXILIOS EN SALUD MENTAL

Propósito de esta guía

Esta guía de primeros auxilios en salud mental para personas con problemas en riesgo de suicidio está destinada a miembros de la comunidad sin ningún tipo de experiencia o estudio previo referido a la salud /salud mental. El rol de la persona capacitada por medio de esta guía es poder ofrecer una ayuda inicial a la persona en riesgo de suicidio hasta cuando ésta reciba la ayuda profesional en el caso de ser necesaria.

¿Cómo se desarrolló esta guía?

Esta guía fue desarrollada en base a la opinión de expertas y expertos de la Argentina y de Chile, tanto en cuanto a su formación profesional (médicos generalistas, psiquiatras, psicólogos, psicólogos sociales, etc.) como personas con experiencia vivida en el tema del riesgo de suicidio (propia o como cuidador/a informal).

El equipo de Argentina y de Chile utilizó las guías de primeros auxilios existentes en Australia y realizó la adaptación correspondiente con el asesoramiento de expertos locales. Los detalles de la metodología empleada pueden consultarse en la publicación Encina E, Rodante D, Agrest M, Tapia-Muñoz T, Zamora-Vidal I, Ardila-Gómez S, Alvarado R, Leiderman E, Reavley N. Development of mental health first-aid guidelines to help persons at suicide risk: a Delphi expert consensus study in Argentina and Chile. (en proceso de publicación).

Cómo utilizar estas recomendaciones

Esta guía reúne un conjunto de orientaciones generales. Cada persona es única y es importante adaptar el apoyo que se le entregue a sus necesidades. En este sentido, puede que haya personas que no sean ayudadas aún siguiendo estas recomendaciones o que éstas no sean apropiadas para algunas personas, aun cuando están formuladas en consideración a aspectos culturales de la población chilena y argentina.

Además, debe considerarse muy especialmente que algunas personas presentan junto con el riesgo de suicidio algún otro tipo de problema de salud o de salud mental que pueden requerir abordajes específicos y especializados.

Las secciones de fondo blanco corresponden a las recomendaciones de los expertos locales. En tanto, los recuadros de fondo anaranjado son agregados realizados por los editores de esta guía para complementar o aclarar las sugerencias de los expertos locales.

Estas guías fueron desarrolladas por la Universidad de Palermo, la Universidad de Chile y la Universidad de Melbourne y están protegidas por el derecho de autor. Sin embargo, se pueden usar libremente si no es con fines de lucro.

Se recomienda citar como: Primeros Auxilios en Salud Mental, América Latina. Brindando ayuda a una persona con riesgo de suicidio: Guía de primeros auxilios en salud mental. Universidad de Chile, Universidad de Palermo y Universidad de Melbourne, 2023.

Esta guía se ha elaborado como parte de un conjunto de directrices sobre la mejor manera de asistir a una persona con problemas de salud mental y está disponible en https://mhfainternational.org/current-mhfa-research/. El conjunto de recomendaciones de primeros auxilios en salud mental en Inglés puede consultarse y descargarse en *https://mhfa.com.au/mental-health-first-aid-guidelines#mhfaesc*

MENTAL HEALTH FIRST AID
Chile - Argentina

CÓMO BRINDAR AYUDA A UNA PERSONA EN RIESGO DE SUICIDIO

GUÍA DE PRIMEROS AUXILIOS EN SALUD MENTAL

Si tiene dudas respecto de su utilización puede dirigirse a:

Demián Rodante, dr.demianrodante@gmail.com
Esteban Encina, esteban2@uchile.cl
Martín Agrest, magrest66@gmail.com
Nicola Reavley, nreavley@unimelb.edu.au. +61 3 9035 7628
